# Metal leakage from orthodontic appliances chemically alters enamel surface during experimental in vitro simulated treatment

**DOI:** 10.1038/s41598-024-56111-4

**Published:** 2024-03-05

**Authors:** Justyna M. Topolska, Agata Jagielska, Sylwia Motyl, Gabriela A. Kozub-Budzyń, Luiza Kępa, Barbara Wagner, Katarzyna Wątor

**Affiliations:** 1 Department of Mineralogy, Petrography and Geochemistry, Faculty of Geology, Geophysics and Environmental Protection, AGH University of Krakow, 30-059 Krakow, Poland; 2 Laboratory of Theoretical Aspects of Analytical Chemistry, Faculty of Chemistry, Biological and Chemical Research Centre, University of Warsaw, 02-089 Warsaw, Poland; 3 Department of Oral and Maxillofacial Surgery, Rydygier Hospital, 31-826 Krakow, Poland; 4 Department of Geology of Mineral Deposits and Mining Geology, Faculty of Geology, Geophysics and Environmental Protection, AGH University of Krakow, 30-059 Krakow, Poland; 5 Department of Hydrogeology and Engineering Geology, Faculty of Geology, Geophysics and Environmental Protection, AGH University of Krakow, 30-059 Krakow, Poland

**Keywords:** Dentistry, Disease prevention, Oral manifestations, Risk factors, Biomineralization, Tissues, Metals, Experimental models of disease

## Abstract

Human enamel is composed mainly of apatite. This mineral of sorption properties is susceptible to chemical changes, which in turn affect its resistance to dissolution. This study aimed to investigate whether metal leakage from orthodontic appliances chemically alters the enamel surface during an in vitro simulated orthodontic treatment. Totally 107 human enamel samples were subjected to the simulation involving metal appliances and cyclic pH fluctuations over a period of 12 months in four complimentary experiments. The average concentrations and distribution of Fe, Cr, Ni, Ti and Cu within the enamel before and after the experiments were examined using ICP‒MS and LA‒ICP‒MS techniques. The samples exposed to the interaction with metal appliances exhibited a significant increase in average Fe, Cr and Ni (Kruskal–Wallis, p < 0.002) content in comparison to the control group. The outer layer, narrow fissures and points of contact with the metal components showed increased concentrations of Fe, Ti, Ni and Cr after simulated treatment, conversely to the enamel sealed with an adhesive system. It has been concluded that metal leakage from orthodontic appliances chemically alters enamel surface and microlesions during experimental in vitro simulated treatment.

## Introduction

Orthodontics is the third most prevalent treatment area within the field of dentistry^1^. While professional orthodontic therapy requires the expertise of an orthodontist, patients take an active part in selecting the type of appliance. Numerous varieties of orthodontic systems are available, ranging from fixed braces equipped with either metal or aesthetic brackets to lingual braces, 3D printed tools, and other rapidly evolving devices^2,3^. Patients commonly base their selection of a treatment system on two primary factors: the aesthetics of their appearance during treatment and the associated cost^4^. Therefore, the quality of alloys and manufacturing of inconspicuous parts such as bands, buttons, ligatures or retainers is not subject to such dynamic development as the quality of visible parts and is not a decisive factor in the choice of therapy system.

The presence of trace amounts of metals in human tooth enamel is a natural state^5,6^. Among other reasons, this is because enamel consists of minerals from the apatite group in more than 90% of its constitution^7^. These minerals have an extremely flexible crystal structure that allows for a wide range of ionic substitutions in their unit cell. The chemical formula of apatite can be written in the general form: *X*_10_(*Y*O_4_)_6_*Z*_2_, where *X* = e.g.: Ca, Pb, Sr, Ba; *Y* = , e.g.: P, As, V, C, S; and *Z* = e.g.: Cl, F, OH, I^8,9^. The abiotic (passive) process of mineralization and demineralization of apatite that builds enamel is a lifelong process^10–12^. Enamel-building minerals equilibrate with surrounding solutions via dissolution and recrystallization. Hence, the elements present in saliva can build into their structure^13–17^. Therefore, the chemical composition of enamel depends not only on genetic factors but also on age, living environment, and individual dietary and hygiene habits^6,7,10,18^. An imbalance in the process of enamel remineralization and demineralization leads to enamel caries^19–21^.

In some orthodontic patients, an elevated amount of enamel demineralization has been noted, manifested in the form of so-called white spots, which, like prisms with excessive mineralization, are considered a predisease stage^22,23^. It has been believed that the direct cause of the formation of plaque and caries in orthodontic patients are the difficulties in maintaining proper hygiene around the brackets and the accumulation of bacteria there^24^. Fluoride therapy and frequent orthodontic control have been proposed to address this problem^25,26^. It has also been reported that certain white spots in orthodontic patients endure for over six weeks^22^ and that fluoride therapy is inefficient on long-existing lesions of this nature^27^.

The metal parts of fixed orthodontic appliances are made of alloys that mainly contain: Fe, Ti, Ni, and Cr. Saliva, food, and beverages, as well as oral hygiene products, affect the condition of orthodontic appliances^28–30^. The process of metal leakage from their parts due to various factors has been widely described^31–33^. Attempts have been made to determine the impact of this phenomenon on human health^34,35^. Nevertheless, minimal research has been conducted to date on the effect of metal leakage from appliances on the chemical composition of tooth enamel. It is not possible to analyse the chemical composition of the patients’ enamel before and after orthodontic treatment. Tooth extraction may be performed before treatment, assuming that teeth undergoing the straightening process perform their function in the oral cavity for as long as possible. This constraint renders it unfeasible to carry out clinical trials that can yield statistically significant data on this subject matter. During a study of the content of Ni in dental plaque, an elevated concentration of this metal was identified in orthodontic patients^36^. It is also known that the substitution of metal ions in enamel-building apatite changes its physicochemical properties, which are important in terms of mineral stability, i.e., enamel health^5,37^. However, evidence that metal orthodontic appliances locally change the enamel chemical composition is missing.

Therefore, the aim of this study was to determine the effect of metal ions leakage from orthodontic appliances on the elemental composition of tooth enamel in a series of in vitro experiments. The null hypothesis tested herein assumed that the presence of metal parts of orthodontic appliances do not significantly affect the chemical composition of enamel during orthodontic treatment simulations, in terms of its Fe, Cr, Ni, and Cu content. This null hypothesis was tested against the alternative hypothesis (H_1_) of a difference.

## Results

In a series of in vitro experiments, 54 healthy molars (divided into 107 enamel samples) were subjected to a simulated orthodontic treatment with a use of metal appliance and involving cyclic pH fluctuations over a period of 12 months. Details of the experimental setup are available in Table 1 and in a comprehensive flow chart presented in Supplementary Fig. S1. The total concentrations of selected metals in enamel samples after Experiments: #1, #2 and #3 were examined using ICP‒MS whereas their distribution before and after Experiment #4 was analysed with a LA-ICP‒MS technique.

**Table 1:** Simplified description of the experimental setup

Experiment	Experimental group	Group label	Reactors	Number of enamel samples ^a^	Appliance set ^b^	Number of solutions pH cycles^c^	Aim of the experiment
#1	Main	1A	1e	12	1	360	The effect of the presence of appliances in solutions on the enamel content of Fe, Cr, Ni, and Cu
			2e	12	1		
			3e^d^	11	1		
	Control	1B	1c	12	0	360	
			2c	12	0		
			3c	11	0		
#2	Main	2A’	1e’	8	0	360	The effect of the solutions’ pH cycles on the enamel content of Fe, Cr, Ni, and Cu
	Control	2B’	1c’	8	0	0	
#3	Main	3A’’	–	10	0	0	The influence of dividing teeth into sample halves on the enamel content of Fe, Cr, Ni, and Cu
	Control	3B’’	–	10	0	0	

^a^1 enamel sample = 1 half of human molar tooth.

^b^Each reactor contained a separate set of orthodontic appliances. The set consisted of: 20 brackets, 4 bands, and 2 archwires (for details see "Orthodontic appliances").

^c^Solution cycle procedure is described in "Solutions and reagents".

^d^In the reactor 3e there was also one additional tooth sample from Experiment #4 ("Spatial distribution of metals in enamel before and after orthodontic treatment simulation (Experiment #4)").

### Total metal concentrations in enamel (Experiment #1, #2 and #3)

Table 2 shows the total concentrations of Fe, Ni, Cr, and Cu in the enamel recorded for the experimental and control groups after termination of Experiments: #1, #2 and #3. To facilitate interpretation, the table also includes a statistical analysis of the results and brief information about the conditions of the experiments. For detailed statistical significance parameters please refer to Supplementary Table S1a–d.

**Table 2:** Total concentrations of selected metals in the enamel samples of Experiments #1, #2 and #3

Experiment		Group	Metal concentration (µg/g)												pH cycles applied	Presence of appliance
			Fe			Cr			Ni			Cu				
			Median	IQR	Sig	Median	IQR	Sig	Median	IQR	Sig	Median	IQR	Sig		
Experiment #1	Main	1A	41.63	30.58	b	1.13	0.87	b	3.05	1.99	b	0.94	0.46	ab	+	+
	Control	1B	12.56	6.68	a	0.31	0.29	a	1.33	0.79	a	1.33	0.96	a	+	−
Experiment #2	Main	2A’	8.28	5.53	a	0.12	0.07	a	0.77	1.28	a	1.20	0.64	ab	+	−
	control	2B’	10.32	4.00	a	0.31	0.34	a	2.47	1.52	ab	0.60	0.41	b	−	−
Experiment #3	main	3A’’	9.17	2.54	a	0.27	0.06	a	0.74	0.12	a	0.55	0.35	b	−	−
	control	3B’’	8.45	1.30	a	0.26	0.05	a	0.67	0.35	ac	0.69	0.35	b	−	−
			*p* < 0.0006*			*p* < 0.002*			*p* < 0.002*			p < 0.003*				

*IQR* interquartile range, *Sig.* significance

Exact *p* value for particular groups are available in Supplementary Materials Table S1a–d.

*Values within a median column followed by different letters in a significance (sig) column differ significantly at given *p.*

Except for the Cu concentration, the results indicate that the concentrations of metals (Fe, Cr, and Ni) in the enamel of the teeth that had contact with the solutions leaching orthodontic appliances (Group 1A) were significantly higher (Kruskal–Wallis, *p* < 002) than in the enamel of the control groups. On the other hand, in the context of metal contents, the composition of the enamel samples that had no contact with metals (reacting in solutions without orthodontic appliance parts) or that were not subjected to pH cycling at all was identical in the vast majority of cases (for particular *p* values please refer to Supplementary Tables S1a–d). It is worth noting that the range of metal concentrations in the samples that were not in contact with the appliance was on average (in µg/g): Fe from 5.79 to 71.31, Ni from 0.20 to 7.15, Cr from 0.06 to 1.71, and Cu from 0.32 to 2.52. However, the enamel that reacted in contact with the metal parts contained on average (in µg/g): Fe from 14.97 to 118.80, Ni from 0.44 to 13.89, Cr from 0.14 to 2.72, and Cu from 0.32 to 2.53. As a result of the conducted experiments, the enamel was enriched to the greatest extent in Fe. Changes in the concentrations of Ni and Cr were significant (Kruskal–Wallis, *p* < 002), but by an order of magnitude lower than the changes in Fe. It is worth mentioning that enamel was not significantly enriched in Cu during the experiments performed (for particular *p* values please refer to Supplementary Tables S1a-1d).

### Spatial distribution of metals in enamel (Experiment #4)

The adopted methodology of research and thorough LA-ICP‒MS analysis allowed for mapping of the distribution of metal concentrations in the enamel before and after the experiments, which simulated orthodontic treatment. Using the LA-ICP‒MS method, the distributions of Fe, Ni, Ti, Cr, and Cu as metals detected in the parts of the orthodontic appliances were analysed.

**Figure 1:**
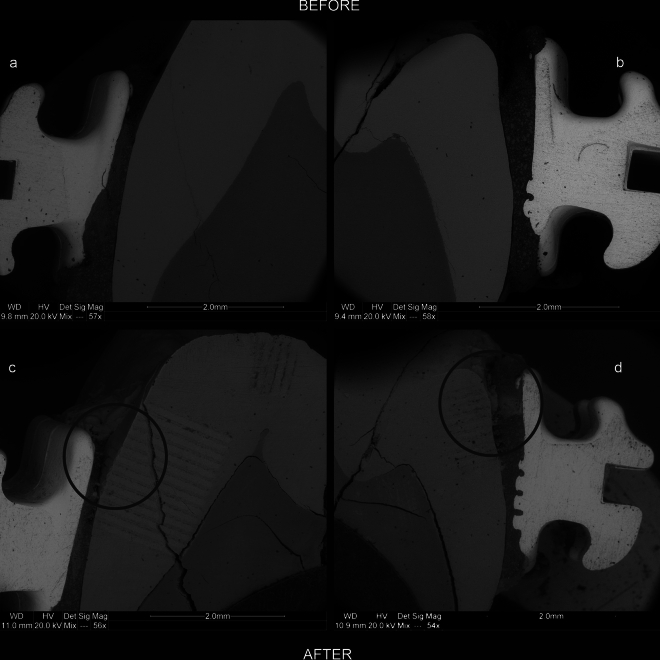
SEM micrographs of the tooth in the lingual–buccal cross-section showing the places where the brackets were attached to the enamel surface. (**a,c)** The lingual part, and (**b**,**d**) show the buccal part. (**a,b**) The state before the experiments, and (**c,d**) show the state after the experiments. The black circles indicate places where the adhesive system has eroded due to the solutions. The lines on the enamel (**c,d**) are LA-ICP‒MS laser beam traces.

**Figure 2:**
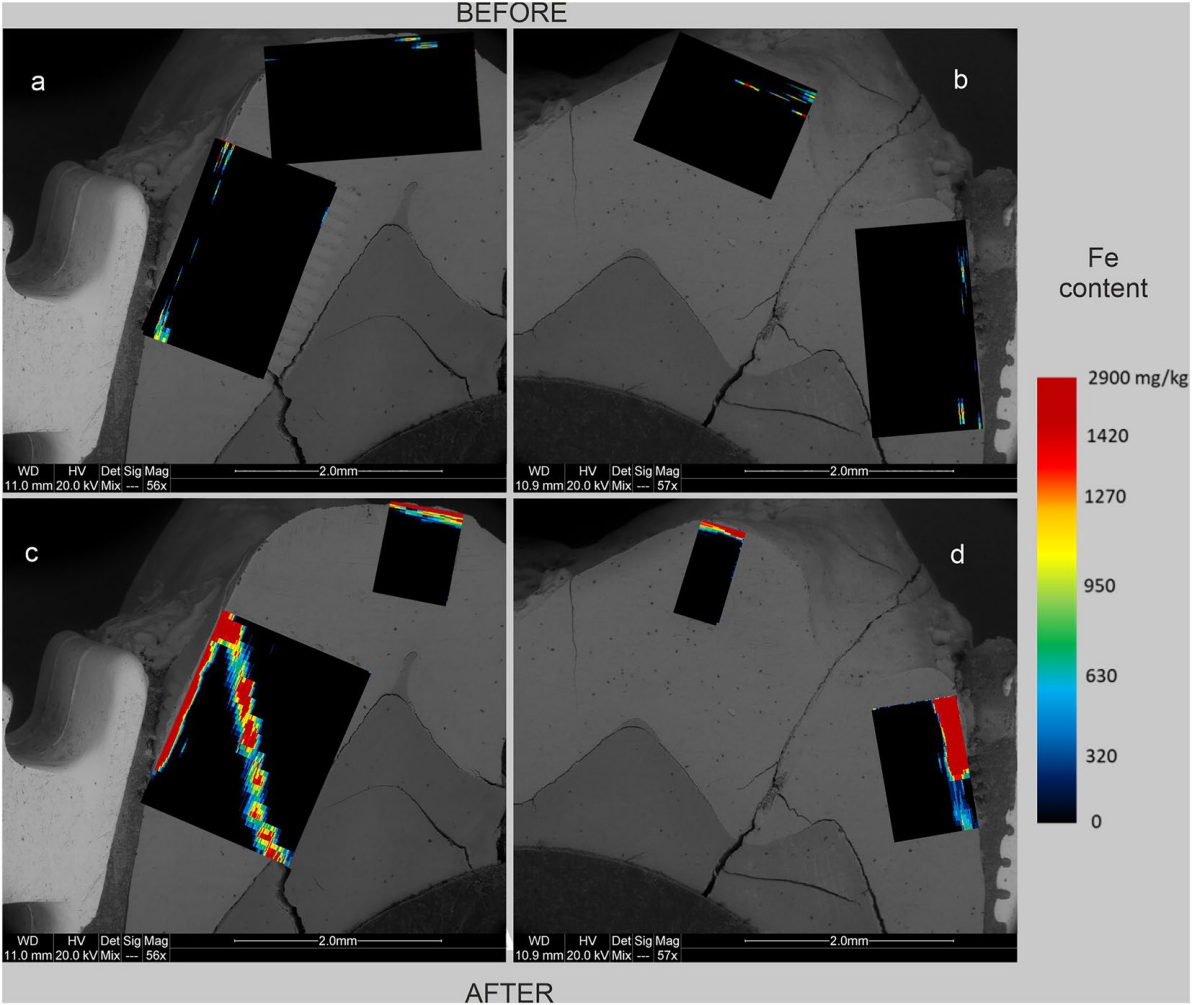
Maps of Ti content in human tooth enamel during laboratory simulation of orthodontic treatment using a fixed metal orthodontic appliance. The maps were made on the basis of LA-ICP‒MS analyses. The tooth is presented in a lingual–buccal cross-section. (**a,c**) The lingual part, and (**b,d**) show the buccal part. (**a,b**) The state before the experiments, and (**c,d**) show the state after the experiments.

**Figure 3:**
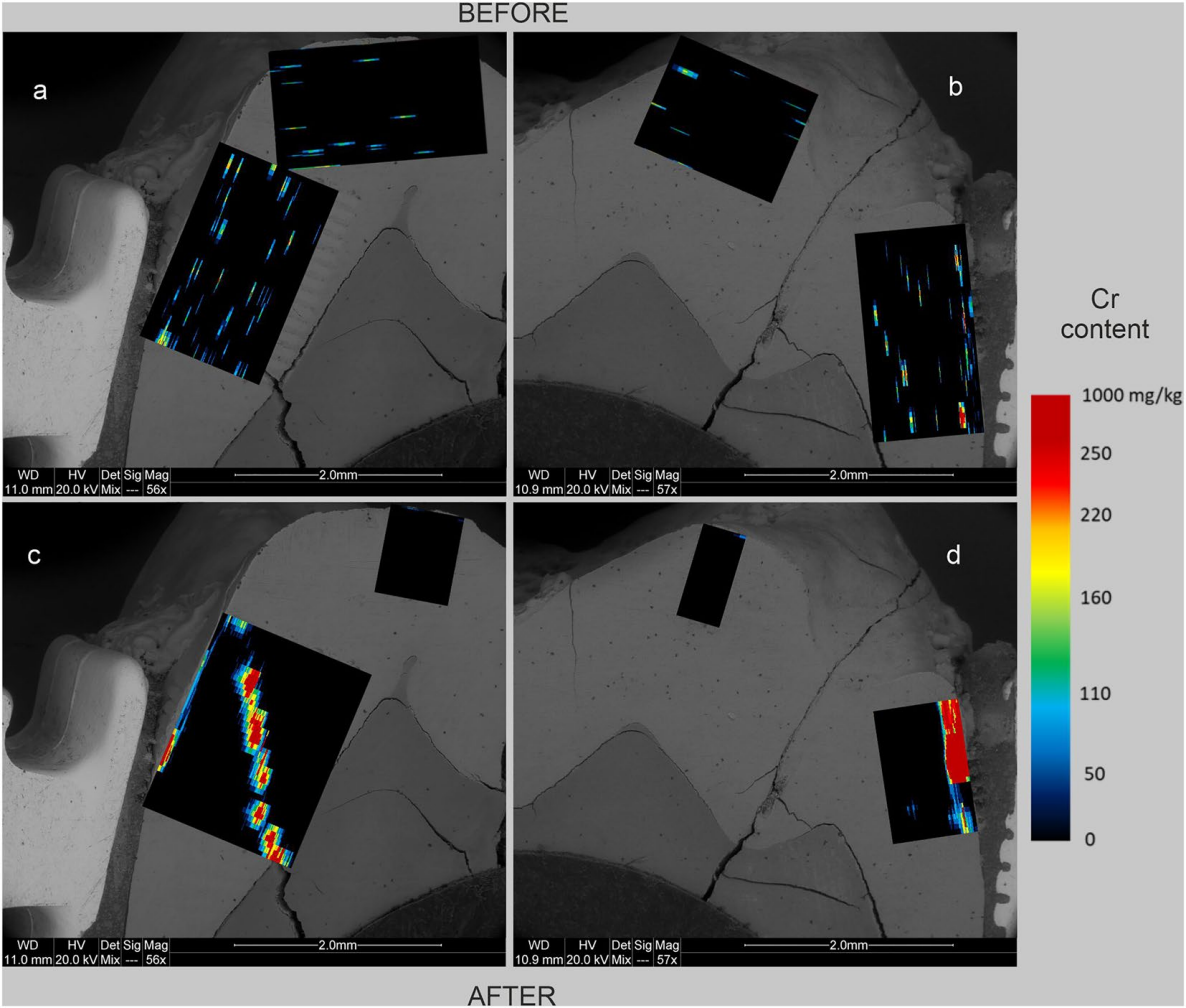
Maps of Fe content in human tooth enamel during laboratory simulation of orthodontic treatment using a fixed metal orthodontic appliance. The maps were made on the basis of LA-ICP‒MS analyses. The tooth is presented in a lingual–buccal cross-section. (**a,c)** The lingual part, and (**b,d**) show the buccal part. (**a,b**) The state before the experiments, and (**c,d**) show the state after the experiments.

**Figure 4:**
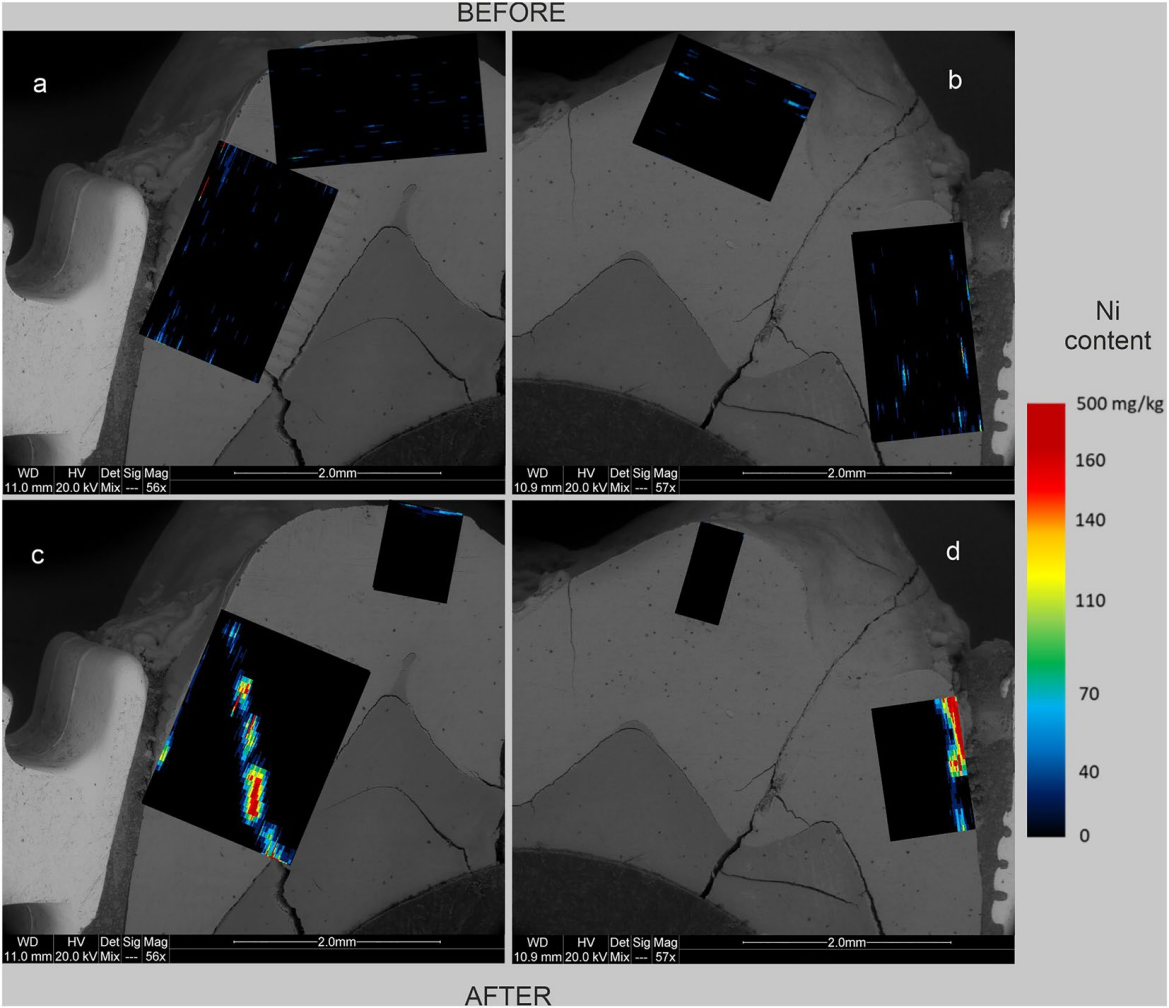
Maps of Cr content in human tooth enamel during laboratory simulation of orthodontic treatment using a fixed metal orthodontic appliance. The maps were made on the basis of LA-ICP‒MS analyses. The tooth is presented in a lingual–buccal cross-section. (**a,c**) The lingual part, and (**b,d**) show the buccal part. (**a,b**) The state before the experiments, and (**c,d**) show the state after the experiments.

**Figure 5:**
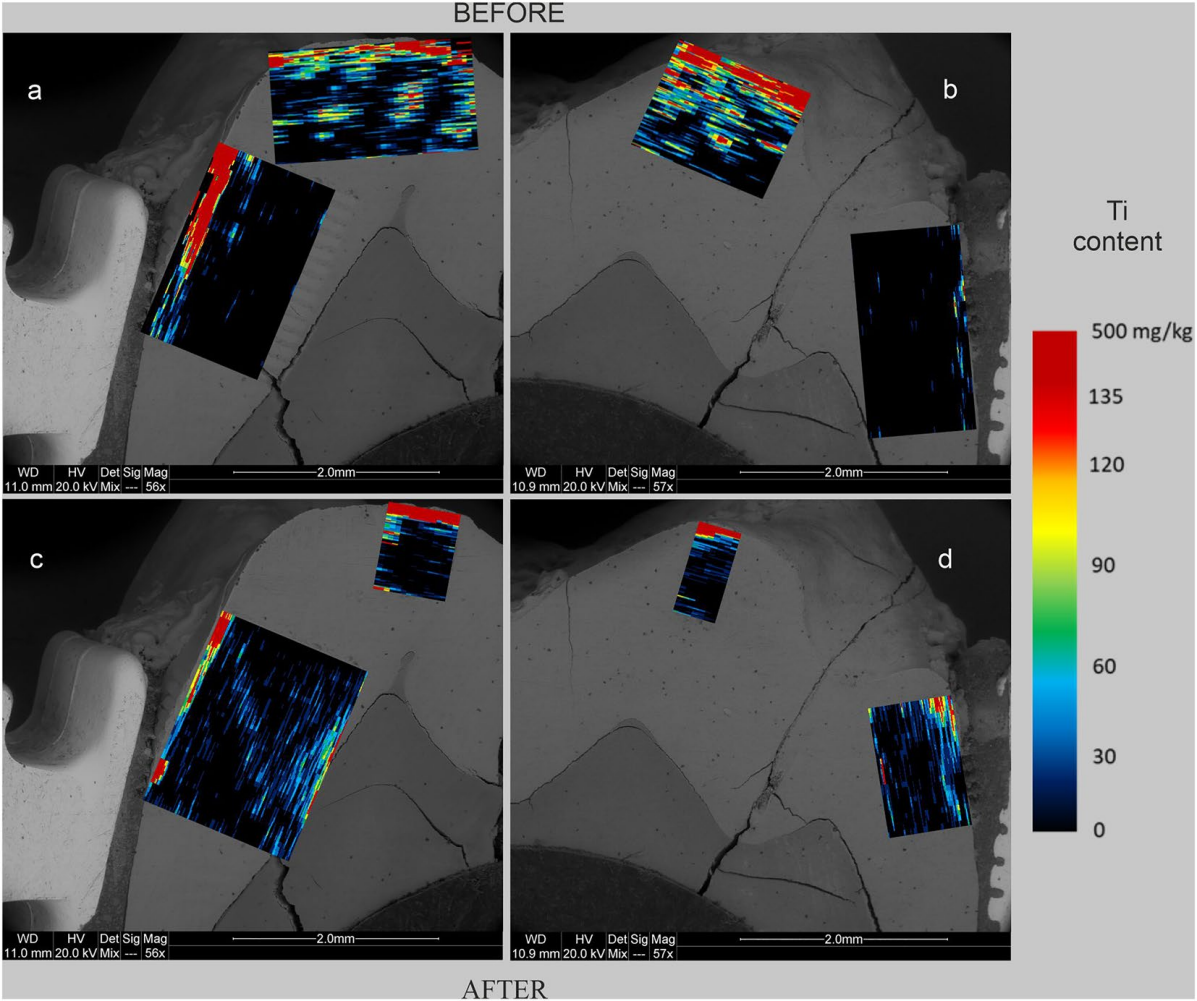
Maps of Ni content in human tooth enamel during laboratory simulation of orthodontic treatment using a fixed metal orthodontic appliance. The maps were made on the basis of LA-ICP‒MS analyses. The tooth is presented in a lingual–buccal cross-section. Figures **a** and **c** show the lingual part, and Figures **b** and **d** show the buccal part. Figures **a** and **b** show the state before the experiments, and Figures **c** and **d** show the state after the experiments.

**Figure 6:**
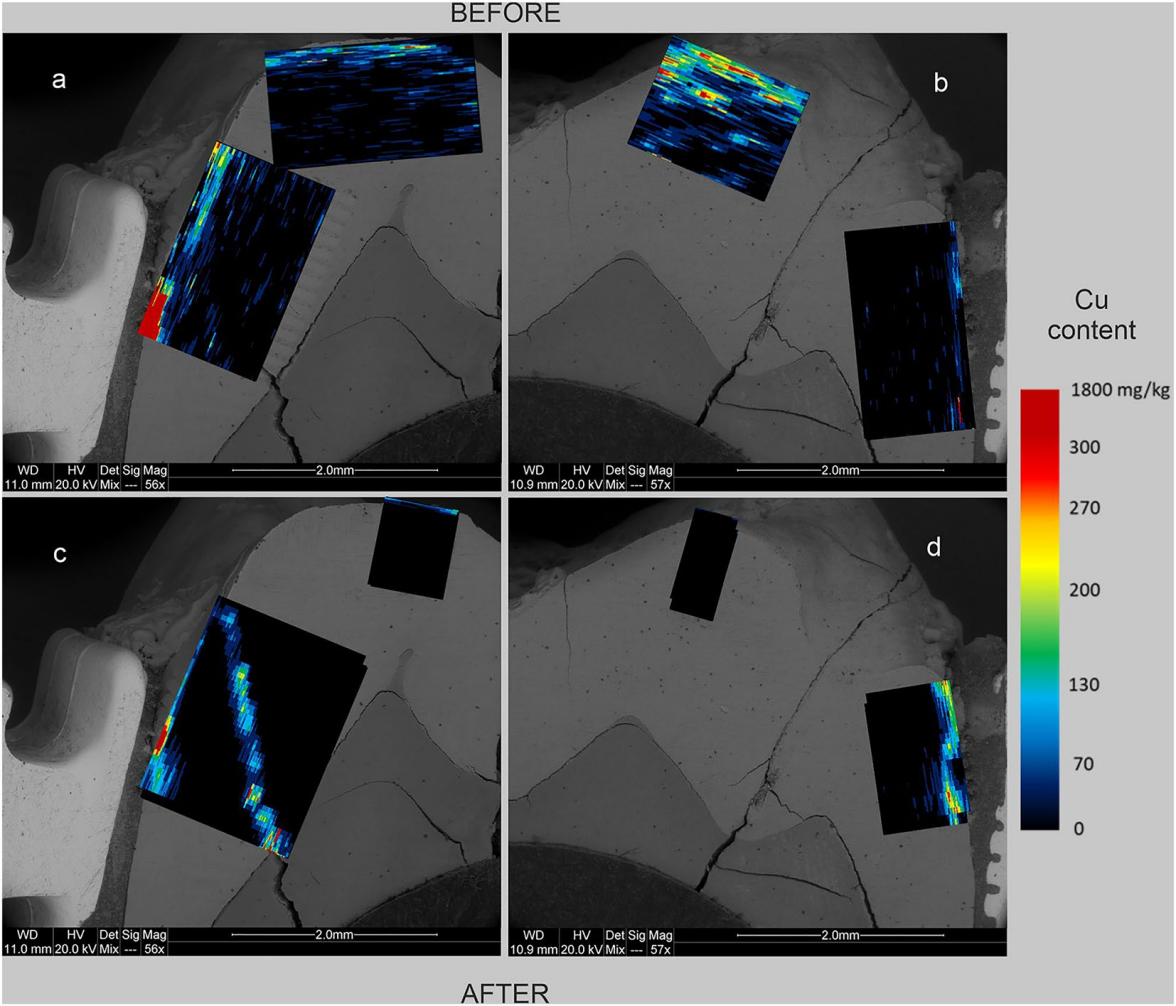
Maps of Cu content in human tooth enamel during laboratory simulation of orthodontic treatment using a fixed metal orthodontic appliance. The maps were made on the basis of LA-ICP‒MS analyses. The tooth is presented in a lingual–buccal cross-section. (**a,c**) The lingual part, and (**b,d**) The buccal part. (**a,b**) The state before the experiments, and (**c,d**) show the state after the experiments.

Figure1a–d present the lingual–buccal cross-section of the tooth before and after pH cycle experiments, showing places where the brackets were attached to the enamel. The presented images indicate that the experiments carried out fatigued the reacting surfaces, deepening the initial tooth cracks, fissures, and defects in the surfaces between the bond, bracket, and enamel. The places under the upper tie wing of the brackets were particularly sensitive in this respect (Fig. 1c, d). In these places, the adhesive system crumbled, creating microfissures where experimental solutions could accumulate. For this reason, these sites were subjected to more LA-ICP‒MS postexperimental analysis than the crown sites. The resulting LA-ICP‒MS maps are shown in Figs. 2, 3, 4, 5, 6. To facilitate interpretation, the maps obtained were plotted on the SEM microphotographs of the tooth sample after the experiments.

The presented results clearly indicate that the conducted experiments influenced the composition of the enamel in terms of metal content. There are differences in the degree of metals assimilation by the enamel depending on the place of analysis and the metal. As shown in Figs. 3, 5, and 6, before the experiments, both the tooth crown and its lateral parts were locally enriched in Ti and to a lesser extent in Cu and Cr. In many places, these metals were present throughout the entire depth of the analysed enamel (up to approximately 1 mm). On the other hand, Fe and Ni were present in the enamel of the examined tooth only locally, in negligible amounts (Fig. 2 and 4). However, in the figures showing the distribution of metals in the enamel after the experiments that simulated orthodontic treatment (Figs. 2, 3, 4, 5, 6), changes in the quantitative composition of metals and local reorganization of their content can be observed.

The outer enamel zone (up to approximately 200 µm) was the most prone to alterations. This zone in the crown of the tooth was enriched primarily in Fe. The concentration of Ti in this part of the enamel maintained its preexperiment value. On the other hand, in the lateral parts of the tooth, the enamel was enriched in Fe, Ni, Cr, and Ti in proximity to the brackets. Particularly noteworthy are the places where the enamel was affected in some way. There was a concentration of metals in the microfissures and cracks, unlike in places where the enamel was effectively sealed by the orthodontic adhesive. These contained comparable amounts of metals before and after the experiments. This is well illustrated in Figs. 2d, 3d, 4d, 5d and 6d, which show the locations of the bracket attachment on the buccal side of the tooth’s cross-section. They showed a sharp border between the place in the enamel effectively sealed by the adhesive system, where the concentrations of metals were close to 0 (black spot), and the places where the adhesive system had been eroded due to the solutions (the highest concentrations).

The presented maps indicate that Fe, Cr, and Ni were distributed in the enamel in a similar manner during the experiments. Ti, on the other hand, maintained its original, discrete distribution over the entire surface of the analysed enamel, although its highest concentrations after the experiments were associated with the outermost enamel zone, fissures, and the interface between the enamel and dentine. It is interesting that after the experiments, the distribution of Cu in the enamel changed, and in the crown part, the amount of this metal even decreased as presented in Fig. 6.

## Discussion

Prisms with altered enamel mineralization in orthodontic patients have been studied for years^22–26^. Individuals subjected to fixed orthodontic treatment are exposed to typical cariogenic factors^19–21^, but it is believed that difficult enamel hygiene and other problems related to the adhesion of brackets may enhance the disease processes^24^. This paper describes, for the first time, another aspect related to orthodontic appliances i.e., the assimilation of metals leaking from the appliance by the enamel. The impact of this phenomenon on post-appliance enamel changes found in some individuals remains unknown.

The results of the conducted research allowed to reject the null hypothesis, which assumed that the presence of metal parts of orthodontic appliances do not significantly affect the chemical composition of enamel during in vitro orthodontic treatment simulations, in terms of its Fe, Cr, Ni concentrations. However, the influence of Cu ions on the chemical composition of enamel has not been recognized as significant and the results obtained for this element confirmed the null hypothesis. It is worth mentioning that Fe, Cr, Ni and Ti are the main components of alloys used for orthodontic purposes, unlike Cu, which is only a trace admixture in some appliances parts. Statistical analysis implied that (i) comparing to the control groups, the enamel exposed to metals leaking from appliance parts was enriched in Fe, Ni, and Cr, and (ii) the adopted experimental technique (procedure of pH cycles and dividing teeth into corresponding halves) did not directly affect the content of these metals in the experimental samples; The results for the group 2A’ were statistically identical to the results for the group 2B’, and the results for the group 1B were statistically identical to the groups 2A’–3B’’.

Influenced by saliva, beverages, and bacteria, the metals from orthodontic appliances are released from the alloys and turn into an ionic form, which allows them to migrate throughout the tooth^31,32,38^. This work clearly demonstrates that metals from parts of orthodontic appliances can be assimilated by enamel during demineralization and remineralization cycles in the range of pH fluctuations typical of the human oral cavity. It is worth noting that the total concentrations of metals (Fe, Cr, Ni, and Cu) both in experimental and control samples were within the ranges typical for human enamel. On the other hand, their spatial distribution locally exceeded the referenced average values^6^. Thus, after orthodontic treatment, enamel that meets the standards at the general, averaged level might locally show different physicochemical properties on a microscopic scale.

It is worth noting that all analysed metal ions can substitute for Ca ions in apatites that build tooth enamel^5,13,39,40^. Some ions in the structure of apatite increase and others decrease the thermodynamic stability of the mineral^16,40^; however, their substitutions always affect the structural parameters of the crystals. Substituted Fe increases the size of the unit cell and reduces mineral crystallinity. Ti substitutions decrease crystallinity and the size of the unit cell, while Ni and Cr increase crystallinity but decrease the size of the unit cell^5^. In thermodynamic predictions, Fe and Ni substituted apatites are less stable than pure Ca-apatite^18^.

As we presented before, stress between the bonds resulting from the heterogeneous structure of the mineral (multiple ion substitutions at the same crystallographic site) may increase the solubility of apatite measured as the release of metal ions occupying the X site in the X_10_(YO_4_)_6_Z_2_ structure^17,41^. Therefore, we deduce that the nonuniformity of enamel caused by the incorporation of metals during orthodontic treatment might result in the release of calcium ions on a microscale. It is not known, whether this may enhance enamel decalcification recognised as a disease stage, therefore we find this issue should be further investigated for responsible development of orthodontic systems.

The dissolution and recrystallization of enamel-building hydroxyapatite is a complex and multistage process^14,42,43^ and the potential assimilation of metals complicates it further. First, the direct substitution of a metal ion for Ca in the apatite structure may change its solubility, leading to the initiation of the enamel decalcification process^14,16,40^. Second, the presence of ions in the saliva may also lead to the precipitation of altered precursor phases for the enamel-building apatite, which in turn may disturb the enamel recrystallization process^44–47^. Finally, the increased number of metal trace elements adsorbed on apatite might promote bacterial biofilm development^38,43,48,49^.

The results show that the assimilation of Ni, Fe, and Cr occurred in the enamel with a similar trend (Supplementary Tab. S2a). On the other hand, Cu did not follow the Fe, Cr, and Ni trends. Similar correlations have been indicated previously^5^. In this study, the experimental enamel samples had their control counterparts in the form of the other half of the same tooth. Using this fact, correlations were made between the enamel metal concentrations after the experiment with a use of appliance and its original composition as described in "Statistics" and presented in Supplementary Table S2b. Interestingly, the greatest dependence in this respect was shown by the postexperimental Fe concentration and the initial amount of Cu in the enamel. The aspect of Cu content in enamel as a marker indicating the potential susceptibility of enamel to assimilation of other metals requires further research.

In this study, it was shown that the outer layer of enamel (up to approximately 200 µm) and the places where it was affected (microfissures) were particularly prone to the assimilation of metal ions. It is known that the outer part of the enamel is the most mechanically resistant: it shows the highest parameters of Young’s modulus of elasticity and hardness. In deeper layers, the enamel becomes less mechanically and chemically resistant^50,51^. Considering that each microfissure may be a potential location for metals leaching from orthodontic devices, the enamel after procedures additionally affecting its structure (e.g., stripping, filling) might be particularly prone to alterations in terms of metal assimilation.

Deterioration of the adhesive system during treatment occurs frequently in orthodontics and is largely due to the physicochemical properties of the adhesive materials^52,53^. The adhesive system under the tie wing of the brackets is particularly prone for degradation. It usually results in a microspace between the brackets and the enamel, so as it was during experiments carried out in this work. These places accumulated the solutions rich in metal ions. In a similar way, metal-enriched saliva can remain in the interdental space (e.g., between the metal band and an adjacent molar or premolar). Microspaces in enamel are a natural place for bacterial biofilm accumulation because maintaining their hygiene is difficult. The increased metal microelements concentration e.g., Fe in these parts of enamel, may additionally support the growth of bacteria colonizing the oral cavity^38,43^.

It has been already known that patients wearing fixed metal orthodontic appliances have an increased number of sulphate-reducing bacteria (SRB) and the presence of stainless steel in the oral environment was indicated as a factor that may have had facilitated the colonisation of SRB^38^. Sulphur reduction and iron oxidation are often coupled in the metabolism of microorganisms. Additionally, the presence of metal trace elements in the enamel may induce bacteria to activate mechanisms enhancing solubilization of the apatite, such as surface colonization and production of organic acids or chelating agents^48,49^. All this, in turn, may lead to the rapid formation of dental plaque or caries.

It is also worth noting that some metal ions e.g.: Cu and Ag or Au are of recognised antibacterial effects. Recently a promising treatment involving Ag-doped resins for tooth lesions restoration has been proposed and widely studied^54^. Leveraging the findings of our study, it is reasonable to infer that materials incorporating elements with antibacterial properties could potentially contribute to dental health during orthodontic treatment. It would be advantageous to develop novel high-quality alloys, particularly those employed in the construction of inconspicuous components within orthodontic systems. Orthodontic bands require special attention in these terms. These parts, usually made of stainless steel, reduce the space between the teeth when applied to molars and are invisible during smiling. Hence, their quality on the one hand is crucial to prevent caries in the interdental spaces, but on the other hand, it may not be substantial for patients.

It should be considered that the absolute content of metals in the enamel after real, not simulated orthodontic treatment may differ from the experimental outcome, presented in this study. The experimental setup and materials used were only an exemplary and simplified model for investigating the so far unrevealed aspects of enamel de-/re-mineralization processes in the presence of a metal orthodontic appliance. The duration of metal-induced, post-appliance changes in the enamel and their contribution to further enamel stability remain uncertain. Presently, the micro-area examination of trace element composition in patients' enamel without causing permanent damage to the teeth is unattainable. Hence, the execution of statistically significant clinical trials investigating whether enamel of individuals subjected to fixed treatment contain an increased amount of trace metals that have leaked from the orthodontic systems is challenging. Consequently, despite the experimental, in vitro nature of our work, the results we present are, to the best of our knowledge, the sole available means of furnishing evidence that orthodontic devices may enrich locally patient’s enamel in metals comprised in their alloys.

## Conclusions

As demonstrated by our in vitro research, metal leakage from orthodontic appliances may potentially influence the elemental composition and chemical homogeneity of enamel surface. During our simulations, the changes within enamel occurred despite employed fluorine treatment and precautions to prevent the formation of bacterial biofilm on the samples. Therefore, it is reasonable to deduce that metal leakage from orthodontic devices may act as one of the agents which induce fluorine treatment-resistant, metal-enriched micro-alterations in enamel surface. The potential synergy effect of this process (involving different alloys and metal-doped materials) with typical cariogenic factors, e.g., enhanced microbial biofilm colonisation, requires further studies and understanding for responsible development of orthodontic systems.

## Methods

### Tooth samples

A total of 54 teeth extracted for orthodontic purposes were used for the experiments. All teeth used in the experiments were undamaged, unbleached molars, with no signs of caries or other diseases. The samples were obtained from nonsmoking women and men aged 25–45 years. Sampling and further experiments’ procedure was approved by Bioethics Committee at the District Medical Chamber in Krakow, Poland (approval number: 154/KBL/OIL/2016). All methods were performed in accordance with the guidelines and regulations provided by the Committee. As agreed, teeth were granted by the Department of Dental Surgery at the University Dental Clinic in Krakow (Poland), after patients’ informed consent. After extraction, the teeth were stored in formalin (10% p. Chempur, Piekary Śląskie Poland). They were then cleaned of soft tissues with 2% papain (Merck, Darmstadt, Germany), deionized water, and fluoride toothpaste. After the samples were thoroughly rinsed, they were dried in a laboratory oven (Heratherm; Thermo Fisher Scientific, MA, USA) for 16 h at 60 °C and stored in this form in a refrigerator (4 °C) until the experiments were conducted. In total, 4 experiments were performed. Details of the experimental setups are presented in "Experiments".

### Orthodontic appliances

Three sets of orthodontic braces were used to conduct the experiments. Each set consisted of the following: 20 brackets, 4 bands (size: 37), and 2 Ni‒Ti or Ni‒Cr archwires. Please refer to Supplementary Table S3 for manufacturing details. The experiments used typical, commercially available, mid-priced sets of braces. The orthodontic archwires were replaced in the reactors every 30 cycles simulating daily changes in oral pH.

### Solutions and reagents

As part of the experiments, the solutions in the experimental reactors were changed daily. The solutions were prepared according to the standard recipe used in pH cycling experiments^56^. Remineralizing solution composition: 1.5 mmol CaCl_2_ × 2 H_2_O p.a. (Chempur, Piekary Śląskie Poland), 0.9 mmol KH_2_PO_4_ p.a. (Chempur, Piekary Śląskie Poland), 130 mmol KCl p.a. (Chempur, Piekary Śląskie Poland), 20 mmol HEPES bufor p.a. (4-(2-hydroxyethyl)-1-piperazine ethane sulfonic acid) (CARL ROTH, Karlsruhe, Germany), and KOH p.a. to adjust pH 7.0 (Chempur, Piekary Śląskie Poland). Demineralizing solution composition: 1.5 mmol CaCl_2_ × 2 H_2_O p.a. (Chempur, Piekary Śląskie Poland), 0.9 mmol KH_2_PO_4_ p.a. (Chempur, Piekary Śląskie Poland), acetic acid p.a. (50 mmol), and KOH p.a. to adjust pH 4.3 (Chempur, Piekary Śląskie Poland).

Fluoride (0.0047 mmol NaF 99+% (Acros Organics, Thermo Scientific Chemical, Delphi, India)), was added to the remineralization solution as it plays a crucial role in both enamel remineralization and metal corrosion^24,25,33^. The solutions were prepared using deionized water with a conductivity of 5 µS/cm produced by a R5Uv device (Hydrolab, Straszyn, Poland). In addition, a commercially available oral hygiene liquid (Elmex; Colgate-Palmolive, Świdnica, Poland), recommended for oral hygiene during orthodontic treatment, was used in the daily pH cycles. The pH of the fluid, measured using a CPI-505 device with an EPS-1 electrode (Emeltron, Zabrze, Poland), was 4.50 ± 0.05.

The daily sequence of the solutions in the reactors was as follows: step 1—demineralization (30–45 min); step 2—hygiene: mouthwash (2 min), tap water (10 s), deionized water (10 s); step 3—remineralization (3–6 h); step 4—demineralization (30–45 min); step 5—hygiene: mouthwash (2 min), tap water (10 s), deionized water (10 s); step 6—remineralization (16–19.5 h). Demineralization and remineralization solutions’ volume was 100 ml, while mouthwash solution volume was 50 ml. The proportions of remineralization and demineralization time were determined by taking into account the abiotic nature of the experiment (lack of enzymes and organic constituents protecting the enamel against dissolution)^42^. The pH cycle has been designed to prevent excessive demineralization of the enamel on the one hand and to maintain the acid shock occurring in the oral cavity, which causes leakage of metals from the appliance on the other hand^25,29^. Daily changes in the concentration of Ca in the experimental solutions resulting from element release (demineralization) or uptake (remineralization) were balanced (Supplementary Fig. S2). On the other hand, the amounts of metals released from the parts of orthodontic appliance (Supplementary Fig. S3) were within the range of previously published data^32^.

### Experiments

#### Total concentrations of metals in enamel

This part of the research consisted of three complimentary experiments: Experiment #1 (the effect of the presence of appliances in the solutions on the enamel content of: Fe, Cr, Ni, and Cu); Experiment #2 (the effect of the applied solutions’ pH cycles on the enamel content of: Fe, Cr, Ni, and Cu); Experiment #3 (the influence of dividing teeth into sample halves on the enamel content of: Fe, Cr, Ni, and Cu). In each experiment, there was a main (experimental) and control group. The details of the experimental setup are presented in Table 1 and in Supplementary Fig. S1. The Experiments #2 and #3 were conducted to ensure that the potential differences in enamel metal content between the main group and the control group in the Experiment #1 were solely a result of the presence of the appliance and not of the experimental procedures or of the inhomogeneous nature of the enamel.

To prepare the experimental samples, 53 out of the 54 extracted and pre-prepared teeth (see "Tooth samples") were cut into corresponding halves labelled as “A”s and “B”s. This resulted in total 106 enamel samples, which were arranged for the Experiment #1, Experiment #2, Experiment #3 and their controls. The “A” tooth halves were used in the main groups, whereas their corresponding “B” halves were arranged as the control samples. Cutting along the lingual–buccal cross-section was performed with a water-cooled dental separator (DFS-DIAMO, Riedenburg, Germany). The exposed tooth cross-sections were secured with stickers (Color Coding Dots 3010; Avery Dennison Zweckform, München-Grünwald, Germany). This method effectively protects the enamel surface in experimental studies using a pH cycle, which has been confirmed previously^55^. The stickers were replaced every 15 solution cycles (for details see below).

The Experiment #1 involved 70 enamel samples. 35 tooth halves (A-s) of the main, experimental group (labelled as: 1A) and 35 corresponding tooth halves (B-s) of the control group (labelled as: 1B) were placed in the 100 ml high-density polypropylene (HDPP) reactors. In total, there were six reactors in this experiment, i.e., three reactors for each group with: 12, 12 and 11 samples in each of them, respectively. Reactors of the experimental group additionally contained orthodontic appliance as indicated in Table 1. The solutions in each reactor were changed daily according to the sequence described in "Solutions and reagents". The reactors were disinfected once a day with disinfectant solution (Pursept A Xpress; Schulke, Norderstedt, Germany) and thoroughly washed with deionized water. The samples were rinsed in deionized and tap water on polypropylene sieves: the contents of a given reactor were placed in the assigned sieve and rinsed under a running stream of deionized or tap water while shaking vigorously. Once a week, the samples were disinfected with disinfectant solution (Pursept A Xpress; Schulke, Germany). This applied procedure allowed long-term experiments to be conducted in a continuous mode without biofilm contamination of reactors, samples, and appliances.

The experiments were conducted for 18 months, during which a total of 360 rinsing cycles were performed. For technical breaks, the samples were placed in a laboratory refrigerator at temperature 4 °C (Whirlpool, Wrocław, Poland) in reactors filled with remineralizing solution to cover the tooth samples^17,29^. This lowered the kinetics of the de and remineralization reactions (Supplementary Fig. S2). During the experiments, the reactors were placed in an incubator at 36.6 °C and shaken at 80 RPM (GLF 3033; Gesellschaft für Labortechnik, Burgwedel, Germany). The reactors were covered with a polypropylene lid, enabling air exchange and protecting against evaporation. After the experiments, the enamel samples were rinsed thoroughly with tap water (2 min) and deionized water (2 min), disinfected with Pursept A Xpress (Schulke, Norderstedt, Germany) solution, and placed in a refrigerator (4 °C) prior to metal concentration analysis using ICP‒MS (details described in "Analytical methods").

The Experiment #2 involved 16 samples. The main, experimental group (labelled as 2A’) involved 8 tooth halves (A’-s), whereas the control group (labelled as 2B’) arranged remaining corresponding 8 tooth halves (B’-s). Samples were placed in the 100 ml HDPP reactors covered with a polypropylene lid. In total, there were two reactors in this experiment, i.e., one for each group with 8 enamel samples in it. Similarly to the group 2B of the Experiment #1, the enamel from the main group (2A’) of the Experiment #2 undergone 360 pH cycles (during 18 months), at 36.6 °C and 80 RPM (GLF 3033; Gesellschaft für Labortechnik, Burgwedel, Germany). The procedures for the solution sequence as well as the maintaining of the tooth samples and the reactors were identical to those in Experiment #1. The samples from the control group (2B’) of the Experiment #2 were placed in the HDPP reactor in a laboratory refrigerator at 4 °C (Whirlpool, Wrocław, Poland) for the whole experimental time (18 months). After the experiments, the enamel samples were rinsed thoroughly with tap water (2 min) and deionized water (2 min), disinfected with Pursept A Xpress (Schulke, Norderstedt, Germany) solution, and placed in a refrigerator (4 °C) prior to metal concentration analysis using ICP‒MS (details described in "Analytical methods").

The Experiment #3, involved 20 samples. The main, experimental group (labelled as 3A’’) consisted of ten tooth halves (A’’-s) and the control group (labelled as 3B’’) arranged corresponding ten tooth halves (B’’-s). The enamel samples from both 3A’’ and 3B’’ groups were rinsed thoroughly with tap water (2 min) and deionized water (2 min), disinfected with Pursept A Xpress (Schulke, Norderstedt, Germany) solution, and placed in a refrigerator (4 °C) prior to metal concentration analysis using ICP‒MS (details described in "Analytical methods"). There was no pH cycling involved in this experiment.

#### Spatial distribution of metals in enamel before and after orthodontic treatment simulation (Experiment #4)

For this experiment, one tooth was prepared differently than the other 53 samples. After washing and cleaning the soft tissues (description in "Tooth samples"), two brackets from the set used in the reactor 3e of Experiment #1 (Table 1) were adhered to the tooth surface on its buccal and lingual sides using the Transbond TM XT kit (3M Unitek, CA, USA). The procedure used was in accordance with the adhesive system manufacturer’s guidelines. Phosphoric acid (36%) (ARKONA, Niemce, Poland) was used as the etching agent.

Then, using sandpaper made of SiC and 1 micron Poly-Top-DUO Diamond slurry (Microdiamant, Lengwil, Switzerland), the lingual–buccal cross-section of the tooth was exposed and analysed at selected sites using a scanning electron microscope with an energy dispersive spectrometer (SEM‒EDS) and a laser ablation inductively coupled plasma mass spectrometer (LA‒ICP‒MS). For analytical details please see "Analytical methods". After the analyses, the exposed cross-section was secured with stickers (Color Coding Dots 3010; Avery Dennison Zweckform, München-Grünwald, Germany). This method effectively protects the enamel surface in experimental studies using a pH cycle and has been confirmed previously^55^. The stickers were replaced every 15 solution cycles.

The tooth was placed in Reactor 3e of the Experiment #1 (Table 1), along with the samples of the 1A group. In this reactor, the tooth underwent 360 cycles of pH changes, similar to the samples of Experiment #1 placed in the same reactor (see "Total concentrations of metals in enamel"). After termination of the pH cycles, the tooth was rinsed thoroughly with tap water (2 min) and deionized water (2 min). The exposed tooth cross-section was polished with 1 micron Poly-Top-DUO Diamond slurry (Microdiamant, Lengwil, Switzerland) to a depth of approximately 50 µm and then cleaned again with tap and deionized water and a cotton swab soaked in concentrated nitric acid (65% ultrapure; Merck, Darmstadt, Germany).

The sample prepared in this way was reanalysed using LA-ICP‒MS at the same sites of the cross-section as before the experiments and then imaged using SEM‒EDS. For analytical details please see "Analytical methods". In total, 12,000 quantitative measurement points were made (Supplementary Table S5). The narrow fissures in the enamel and its contact points with the adhesive system and metal parts of the appliance were analysed particularly thoroughly.

### Analytical methods

Imaging of the dental samples and analysis of the alloy composition of the parts of the orthodontic appliances were performed using a variable pressure field emission scanning electron microscope coupled with an FEI Quanta 200 FEG energy dispersive spectrometer (Thermo Fisher Scientific, OR, USA) at 20 kV (SEM‒EDS).

The analysis of the total metal concentrations in the enamel was performed using ICP‒MS with an iCAP RQ (C2) instrument (Thermo Fischer Scientific, OR, USA) after complete dissolution of 0.05 g of enamel in HNO_3_ acid (65% ultrapure; Merck, Darmstadt, Germany ), followed by dilution with deionized water at a ratio of 1:14. Prior to dissolution, the enamel from each tooth sample was separated from the dentin using a dental drill (#LOT: 582703; Emil Lange Zahnbohrefabrik, Engelskirchen, Germany) and then washed with deionized water and a cotton swab soaked in concentrated HNO_3_ (65% ultrapure, Merck, Darmstadt, Germany) to remove residual fine drill debris and nonenamel parts. The enamel from each sample was then ground with a jade mortar and pestle, averaged using the cone method, and weighed. An NIST SRM 1400 standard (Sigma–Aldrich, Merck, Steinheim am Albuch, Germany) was used as reference material in the analysis. Ti was not analysed in this experiment for technical reasons—there were isobaric and polyatomic interferences during ICP‒MS determination of titanium caused by the presence of high concentrations of Ca and P in the analysed solutions. Supplementary Table S4 shows the detection limits for the analysed elements.

The analysis of the spatial distribution of metals in the enamel was performed using LA‒ICP‒MS. For this purpose, an ICP‒MS NexION 300 spectrometer (Perkin Elmer, MA, USA) coupled with an LSX-213 laser ablation system (CETAC, NE, USA) was used. The operating parameters of the ICP‒MS measurement system and laser ablation conditions are listed in Supplementary Table S6. NIST SRM 1400 and NIST SRM 610 (Sigma–Aldrich, Merck, Steinheim am Albuch, Germany) were used as reference materials. The selection of the reference material for the quantitative analysis of enamel using the LA-ICP‒MS method was described in our previous work^56^. Additionally, the method of making maps of element distribution has been described in previous publications^57–59^. Supplementary Table S7 shows the detection limits for the analysed elements.

### Statistics

TIBICO Statistica version 13.3. and 14.0.0.15 (TIBICO, Palo Alto, CA, USA) was used for all statistical analyses in this study. The results of the metal concentrations in the powdered enamel samples were tested for a normal distribution using the Shapiro–Wilk test, which denied a normal distribution. Therefore, the nonparametric multiple comparisons (2-tailed) of independent variables based on the Kruskal–Wallis test and median tests were used for further interpretation.

The results of the Experiment #1 were tested for correlations between the total concentrations of particular metals in enamel samples, using the Spearman’s rank order correlation tool, with a significance level of p < 0.050. The correlations between the concentrations of Fe, Ni, Cr and Cu in the samples of the group 1A (Experiment #1) have been tested as well as the trends in metals content between groups 1A and 1B of the Experiment#1. Results were presented in Supplementary Tables S2a and S2b.

### Statement of ethics

This study protocol was reviewed and approved by the Bioethics Committee at the District Medical Chamber in Krakow, approval number [154/KBL/OIL/2016]. The Department of Dental Surgery at the University Dental Clinic in Krakow supervised the study participants in terms of the collection of their teeth for the experiments. Teeth were extracted for orthodontic purposes after written informed consent was obtained.

### Supplementary Information

Supplementary Information.

## Data Availability

The data underlying this article are available in the article and in its online supplementary material.
