# Supplementary material for: Metal leakage from orthodontic appliances chemically alters enamel surface during experimental in vitro simulated treatment
Source: Sci Rep. 2024 Mar 5;14:5412. doi: 10.1038/s41598-024-56111-4 (PMC10914722; doi:10.1038/s41598-024-56111-4)
Supplement: Supplementary file 1 — Supplementary Information. [file 41598_2024_56111_MOESM1_ESM.pdf]

Metal leakage from orthodontic appliances chemically alters enamel surface during experimental in vitro simulated treatment

Justyna M. Topolska, Agata Jagielska, Sylwia Motyl, Gabriela A. Kozub-Budzyń, Luiza Kępa, Barbara Wagner, Katarzyna Wątor

Supplementary materials

1. **Experimental set-up** consisted of four complimentary in vitro experiments is presented below.

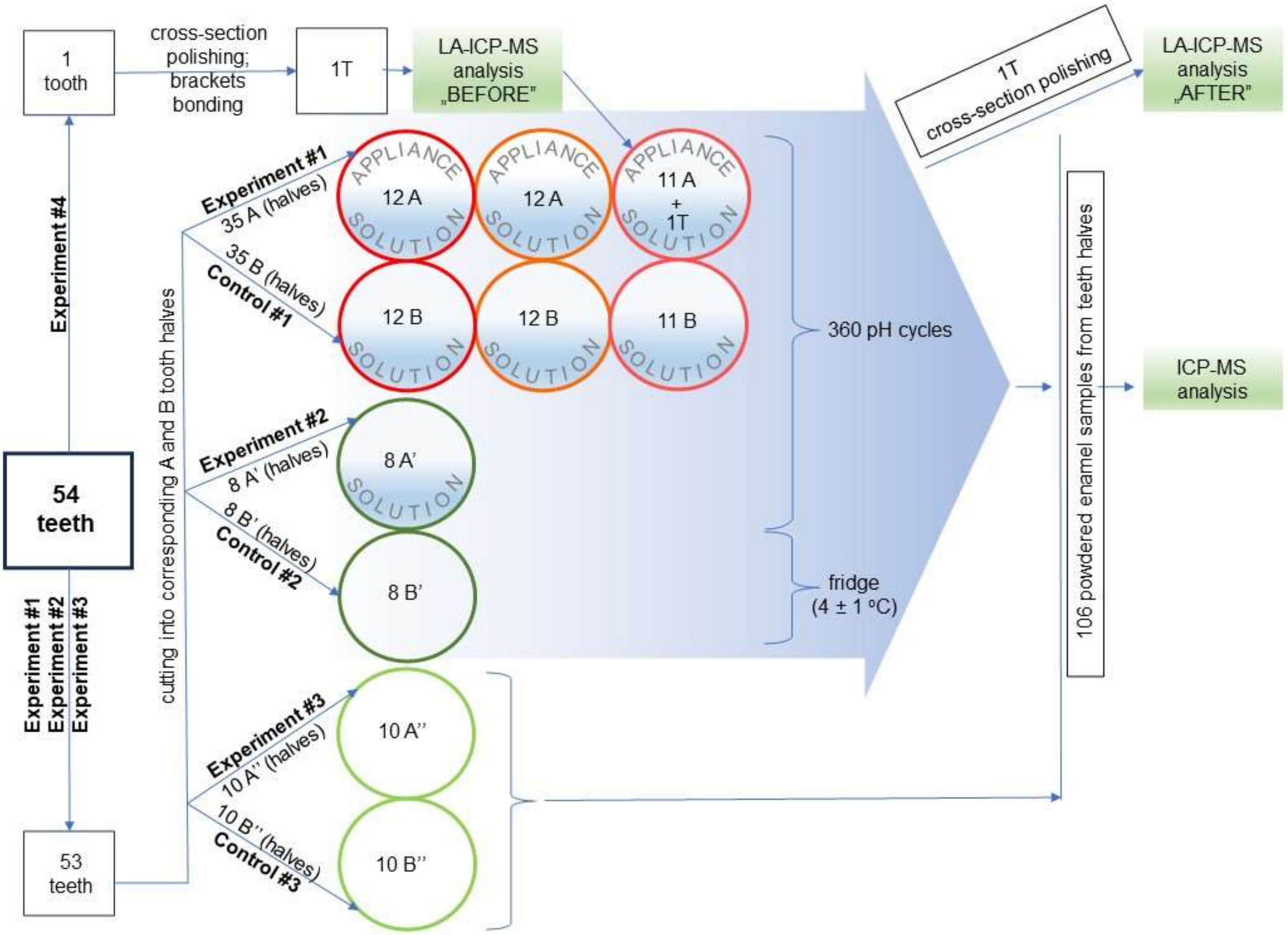

Figure S1. Flow chart providing details of experiments carried out.

2. Statistical analysis of differences between the experimental groups in terms of total metal concentrations in the enamel samples after the Experiments #1, #2, #3. Tables presented below indicate Multiple Comparison *p* values for Kruskal Wallis test: H (df = 5, n = 106).

Table S1a. Analysis for the Fe concentration

	Multiple comparison *p* values (2 tailed) between experimental groups for **Fe concentration [µg/g]** in enamel samples after experiments.					
	Experiment #1 **Group 1A**	Control #1 **Group 1B**	Experiment #2 **Group 2A’**	Control #2 **Group 2B’**	Experiment #3 **Group 3A”**	Control #3 **Group 3B”**
Experiment #1 **Group 1A**		0.000005	0.000009	0.000559	0.000002	0.000000
Control #1 **Group 1B**	0.000005		0.903432	1.000000	0.849195	0.105426
Experiment #2 **Group 2A’**	0.000009	0.903432		1.000000	1.000000	1.000000
Control #2 **Group 2B’**	0.000559	1.000000	1.000000		1.000000	1.000000
Experiment #3 **Group 3A”**	0.000002	0.849195	1.000000	1.000000		1.000000
Control #3 **Group 3B”**	0.000000	0.105426	1.000000	1.000000	1.000000	
p values < 0.050 are significant						

Table S1b. Analysis for the Cr concentration

	Multiple comparison *p* values (2 tailed) between experimental groups for **Cr concentration [µg/g]** in enamel samples after experiments.					
	Experiment #1 **Group 1A**	Control #1 **Group 1B**	Experiment #2 **Group 2A’**	Control #2 **Group 2B’**	Experiment #3 **Group 3A”**	Control #3 **Group 3B”**
Experiment #1 **Group 1A**		0.000008	0.000000	0.002331	0.000181	0.000025
Control #1 **Group 1B**	0.000008		0.078469	1.000000	1.000000	1.000000
Experiment #2 **Group 2A’**	0.000000	0.078469		1.000000	1.000000	1.000000
Control #2 **Group 2B’**	0.002331	1.000000	1.000000		1.000000	1.000000
Experiment #3 **Group 3A”**	0.000181	1.000000	1.000000	1.000000		1.000000
Control #3 **Group 3B”**	0.000025	1.000000	1.000000	1.000000	1.000000	
p values < 0.050 are significant						

Table S1c. Analysis for the Ni concentration

	Multiple comparison *p* values (2 tailed) between experimental groups for **Ni concentration [µg/g]** in enamel samples after experiments.					
	Experiment #1 **Group 1A**	Control #1 **Group 1B**	Experiment #2 **Group 2A’**	Control #2 **Group 2B’**	Experiment #3 **Group 3A”**	Control #3 **Group 3B”**
Experiment #1 **Group 1A**		0.000107	0.001588	0.002331	0.000037	0.000001
Control #1 **Group 1B**	0.000107		1.000000	1.000000	1.000000	0.228211
Experiment #2 **Group 2A’**	0.001588	1.000000		0.439621	1.000000	1.000000
Control #2 **Group 2B’**	1.000000	1.000000	0.439621		0.118185	0.021188
Experiment #3 **Group 3A”**	0.000037	1.000000	1.000000	0.118185		1.000000
Control #3 **Group 3B”**	0.000001	0.228211	1.000000	0.021188	1.000000	
p values < 0.050 are significant						

Table S1d. Analysis for the Cu concentration

	Multiple comparison *p* values (2 tailed) between experimental groups for **Cu concentration [µg/g]** in enamel samples after experiments.					
	Experiment #1 **Group 1A**	Control #1 **Group 1B**	Experiment #2 **Group 2A’**	Control #2 **Group 2B’**	Experiment #3 **Group 3A”**	Control #3 **Group 3B”**
Experiment #1 **Group 1A**		0.082096	1.000000	0.658288	0.232734	0.409776
Control #1 **Group 1B**	0.082096		1.000000	0.003114	0.000291	0.000740
Experiment #2 **Group 2A’**	1.000000	1.000000		0.294076	0.130111	0.206386
Control #2 **Group 2B’**	0.658288	0.003114	0.294076		1.000000	1.000000
Experiment #3 **Group 3A”**	0.232734	0.000291	0.130111	1.000000		1.000000
Control #3 **Group 3B”**	0.409776	0.000740	0.206386	1.000000	1.000000	
p values < 0.050 are significant						

3. **Correlation coefficients** between total metal concentrations in enamel samples from the Experiment #1.

Table S2a. Correlation coefficients between total Fe, Cr, Ni and Cu concentrations in the enamel samples from the 1A group (Experiment #1).

**Element**	**Fe**	**Cr**	**Ni**	**Cu**
**Fe**	1.00	0.81*	0.60*	0,17
**Cr**	0.81*	1.00	0.51*	0.10
**Ni**	0.60*	0.51*	1.00	0.17
**Cu**	0.17	0.10	0.17	1.00

*Indicated values are significant at $p < 0.050$ (Spearman Rank Order Correlations).

Table S2b. Correlation coefficients between total “final” and “initial” metal concentration in the enamel samples from the Experiment #1*

**Element**		**Element**			
	**Final \ Initial**	**Fe**	**Cr**	**Ni**	**Cu**
	**Fe**	0.41**	0.64**	0.67**	0.79**
	**Cr**	0.31**	0.60**	0.50**	0.84**
	**Cu**	0.28**	0.10	0.44**	0.01
	**Ni**	0.05	0.35**	0.15	0.48**

* Final and initial metal concentration was measured using corresponding halves A and B of the experimental tooth samples (Groups #: 1A and 1B in Experimental setup). Total metal concentrations measured for the control experimental group (Group 1B) was considered as initial metal content in enamel. The enamel in these samples wasn’t reacting with the appliance while it was of the same teeth as the enamel from experimental Group 1A.
** Indicated values are significant at $p < 0.050$ (Spearman Rank Order Correlations).

4. Selected parameters of the orthodontic appliances used in the experiments

Table S3. Selected parameters of the orthodontic appliances used in the experiments

**Part of orthodontic appliance**	**Quantity per set**	**Manufacturer**	**Batch Number**	**Average metal content [wt%]***				
				**Fe**	**Cr**	**Ni**	**Cu**	**Ti**
Bracket	20	Orthoclassic OR, USA	901.2032. LOT#072717	67.36 ± 1.63	15.82 ± 0.58	5.35 ± 1.67	3.60 ± 0.58	-
Band	4	Orthodontic Design and Production, CA, USA	MSDS-054	64.08 ± 1.63	17.40 ± 0.39	7.60 ± 0.34	-	-
Archwire #1	1	Orthoclassic OR, USA	61.40.580.01725 0.017x0.25/0.43x0.64 LOT#302127	57.46 ± 2.65	15.82 ± 0.58	7.11 ± 0.22	-	-
Archwire #2	1	Orthoclassic OR, USA	61.40.520.02125 0.021x0.25/0.53x0.64 LOT#071111	-	-	64.61 ± 3.24	-	35.33 ± 2.16

* Metal content expressed in weight percent was determined using the scanning electron microscopy/energy dispersive X–ray spectroscopy (SEM/EDS) method (description in section 4.5).
± total analytical error represented as standard deviation (SD) from ≥ 5 measurements taken at different locations of the test sample

5. Fluctuation of selected elements in solution during pH-cycle.

One cycle of pH change consisted of (100ml solution volume): Demineralization (30 - 45 min); Hygiene: Mouthwash (2 min.), Tap Water (10 sec.), Deionized Water (5 sec.); Remineralization (3 - 6 h); Demineralization (30 - 45 min.); Hygiene: Mouthwash (2 min.), tap water (10 sec.), deionized water (10 sec.); Remineralization (16 - 19.5 h). After demineralization and remineralization steps, the solutions from the reactors of the Experiment #1 were sampled, filtered using MCE membrane filters of 0.45 µm pore size and analysed using ICP–OES with an Optima 7300 DV (Perkin Elmer, MA, USA) or ICP–MS with an iCAP RQ (C2) (Thermo Fischer Scientific, OR, USA). The results are presented in Figures S2 and S3.

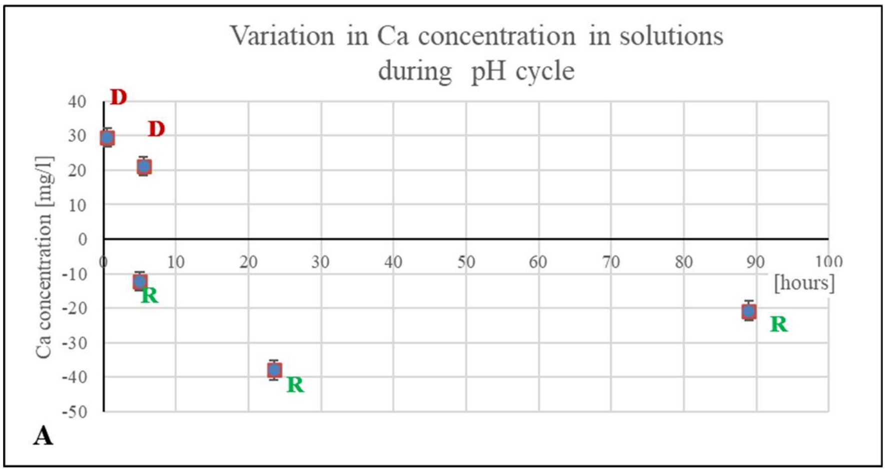

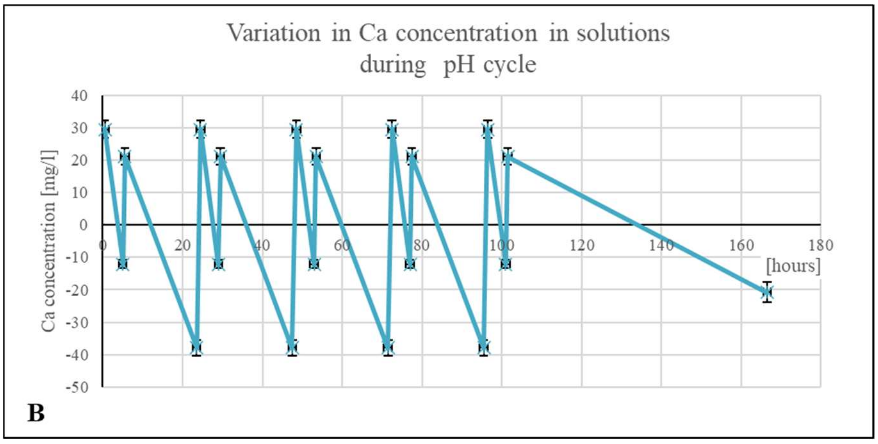

Figure S2. Fluctuation of Ca in solutions during pH cycle. After each step of pH cycle, the solution was sampled and analysed using ICP–OES method, LOQ for Ca was equal to 1 mg/l. Results are presented as the increase and decrease in Ca concentration when compared to the initial level in remineralizing or demineralizing solution (marked in plot as 0). Figure A. Overall changes in Ca during 24 h were equal to ~ 0. Placing the reactors in refrigerator for technical brakes (time point at 89 h) resulted in lowering the rate of the Ca uptake during remineralization step. Figure B. A model of weekly Ca uptake in experimental solutions. Error bars represent standard deviation of experimental triplicates. In general, the remineralization processes slightly outweighed the demineralization processes.

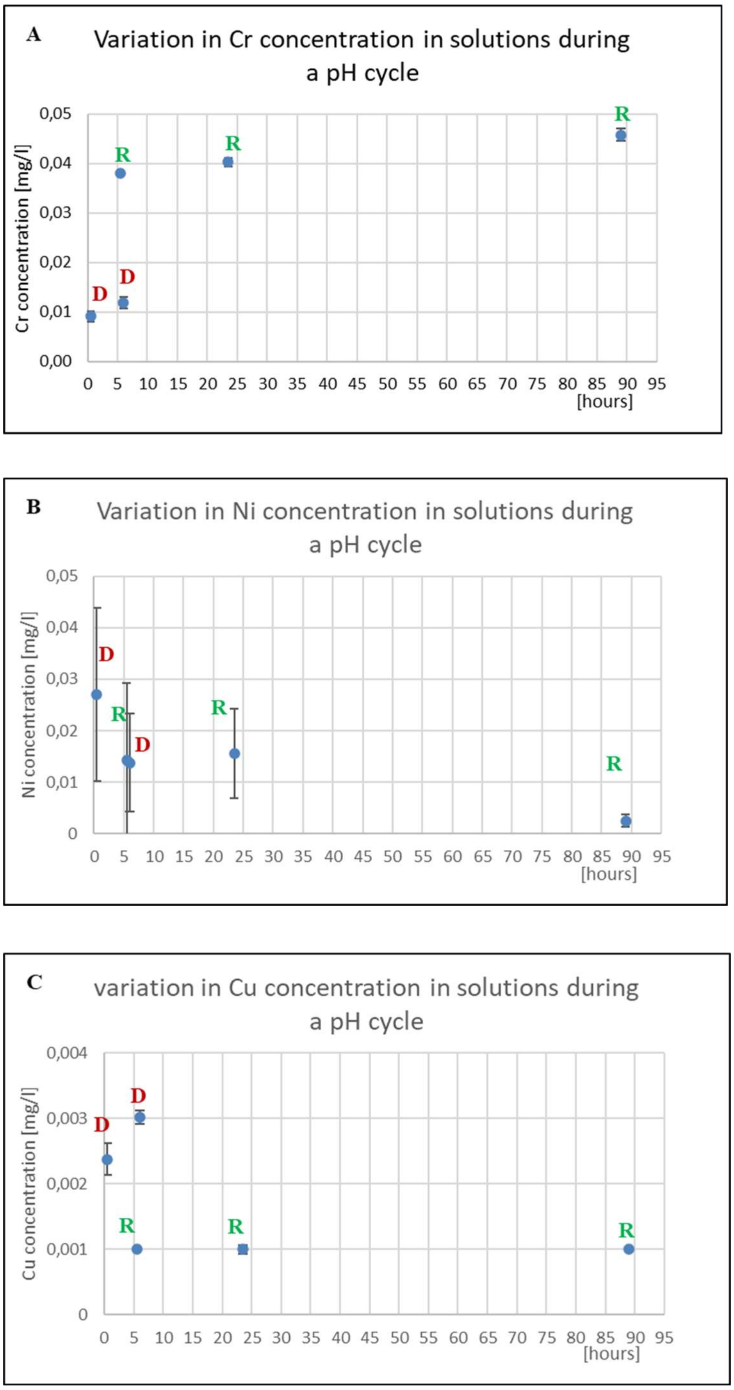

Figure S3.
Fluctuation of selected metals in solutions during pH cycle. A) Fluctuation of Cr; B) Fluctuation of Ni; C) Fluctuation of Cu. Fe concent was below quantification limit i.e., < 0.01 mg/l. Error bars represent standard deviation of experimental triplicates. The results obtained are in accordance to previous reports (32 and literature therein). Some minor differences result most probably from different experimental setups.

3. ICP–MS analysis.

Table S4. Quantification limits (LOQ) for selected elements by ICP–MS

Element	LOQ (mg/l) *
Ni	0.001
Cr	0.005
Fe	0.01
Cu	0.001

4. LA–ICP–MS analysis.

Table S5. Number of measurement points performed using the LA–ICP–MS technique.

Place of analysis	Total number of quantitative measuring points	
	Before *	After **
Bracket area (lingual part)	2240	936
Bracket area (buccal part)	2100	2926
Crown area (lingual part)	1260	423
Crown area (buccal part)	2450	665

* before the experiments (number of pH cycles = 0)
** after the experiments (number of pH cycles = 360)

Table S6. Operating parameters of the ICP–MS measurement system and laser ablation conditions

Plasma power	1200 W
Carrier gas flow / Ar	0,9 L/min
Signal intensity reading time for one isotope	10 ms
Laser wavelength	213 nm
Laser energy	5 mJ
Laser beam diameter	100 µm
Laser operating frequency	20 Hz
Speed of the sample movement relative to the laser beam	15 µm/s
Method of ablation	linear ablation

Table S7. Quantification limits (LOQ) for selected elements by LA–ICP–MS.

Element	Oxide	LOQ (wt% oxide)
Ni	NiO	0.0014
Ti	TiO_2	0.0007
Cr	Cr_2O_3	0.0076
Fe	Fe_2O_3	0.0660
Cu	CuO	0.0003
